# Retraction: Protective effects of calorie restriction and 17-β estradiol on cardiac hypertrophy in ovariectomized obese rats

**DOI:** 10.1371/journal.pone.0358279

**Published:** 2026-09-15

**Authors:** The PLOS One Editors

Following the publication of this article [1] concerns were raised about results presented in Figs 6 and 7. Specifically,

- The following panels appear to partially overlap:
  - Fig 6A Sham+SD and Fig 6A OVX+SD
  - Fig 7A HFD+Oil and Fig 7A HFD+CR+Oil
- Unusually repetitive features were identified in the following panels:
  - Fig 6A Sham+SD+CR
  - Fig 7A SD+CR+E2
  - Fig 7A HFD+CR+E2

The authors’ response did not resolve these concerns.

In light of the above concerns, which call into question the reliability and integrity of the published results, the *PLOS One* Editors retract this article.

ZH, MK, FDM, ARA, and MAB did not agree with the retraction. SD either did not respond directly or could not be reached.
